# Spinal cord ischemia after transcatheter artery chemoembolization for hepatocellular carcinoma: A case-report

**DOI:** 10.1016/j.ijscr.2023.108258

**Published:** 2023-04-24

**Authors:** Le Trung Hieu, Le Van Thanh, Vu Van Quang, Dang Kim Khue, Nguyen Hoang Ngoc Anh, Dao Duc Tien

**Affiliations:** a Hepatobiliary and Pancreatic Surgery Department, 108 Military Central Hospital, Hanoi 100000, Viet Nam; b College of Health Sciences, VinUniversity, Hanoi 100000, Viet Nam; c Department of Gastroenterology, 175 Military Hospital, Ho Chi Minh City 700000, Viet Nam

**Keywords:** Hepatocellular carcinoma, Transarterial chemoembolization, Spinal cord ischemia

## Abstract

**Introduction:**

Transarterial hepatic chemoembolization (TACE) has been used to treat unresectable hepatocellular carcinoma and has gained widespread acceptance as a treatment for both primary and secondary hepatic malignancies.

**Case report:**

We report a case of 78-year-old male patient with chronic hepatitis B, diagnosed with HCC. He underwent the second TACE, and right after the procedure, the patient abruptly developed bilateral lower extremities motor weakness and sensory impairment below the T10 dermatome. Spinal magnetic resonance imaging showed T2-weighted scans showed increased intramedullary signal strength at the T1-T12 level. The patient received supportive care, ongoing rehabilitation, and steroid pulse therapy. The motor strength remained unchanged, but the sensory deficiencies practically disappeared.

**Clinical discussion:**

The hepatic artery injury or decreased flow at the prior TACE site, which causes collateral recruitment, can explain why spinal cord injury following TACE typically happens after the second or third session. It can occasionally result from accidental embolized spinal branches originating from intercostal or lumbar collateral arteries. In our case, we hypothesize the embolism caused the infarction to the spinal cord travel through the connection between the lateral branches of the right inferior phrenic artery and the intercostal arteries, which supply the spinal cord through the anterior spinal artery.

**Conclusions:**

TACE in rare case can have severe complications. A tailored therapeutic strategy, including consideration of a shunt and selection of the vessels utilized for the Lipiodol infusion prior to TACE, is crucial to achieving an optimal end outcome to avert these significant consequences.

## Highlights

• Hepatocellular carcinoma is now leading of cancer-related death in the world.
• Transarterial chemoembolization is the treatment of choice for intermediate HCC stage, and relatively safe.
• We report a rare complication after TACE with spinal cord ischemia.

## 1 Introduction

Hepatocellular carcinoma (HCC) is the third cause of cancer-related death and the fifth most prevalent malignancy worldwide. Only 30 %–40 % of HCC patients receive a diagnosis at an advanced stage for curative treatment [1]. Transarterial hepatic chemoembolization (TACE) is indicated for locally advanced cases, and was widely recommended in guidelines. TACE is also used to downstage beyond transplant criteria tumors. While considered a generally safe procedure, TACE may be accompanying post-embolization syndrome, also known as fever, right upper quadrant pain, nausea, and transaminitis. According to reports, 2.9 % of TACE patients experience complications [2], [3], [4]. The most common cause of neurological impairments after TACE is cerebral embolism of iodized oil (Lipiodol) verified by aberrant imaging findings. We hereby report a rare complication after TACE with spinal infarction. This case presentation follows SCARE guideline [5].

## 2 Case report

**Fig. 1:**
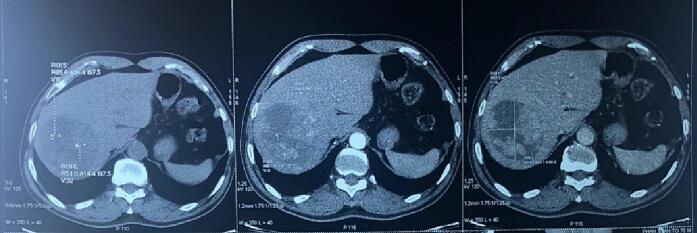
A single 8 cm right hepatic lobe tumor.

The 76-year-old male patient presented with pain in the upper right quadrant one month before being admitted to our center. He has medical history of chronic hepatitis B and hypertension. The CT-Scan revealed an 8-cm-tumor on the right liver with characteristics of HCC. His Alpha Fetoprotein level was 5000 UI/l, others laboratory tests are within normal limit. Due to his age and tumor size and not enough remnant liver volume, we indicated and performed TACE for the patient. He underwent the first TACE uneventfully and was discharged after three days (Fig. 1).

**Figs. 2, 3:**
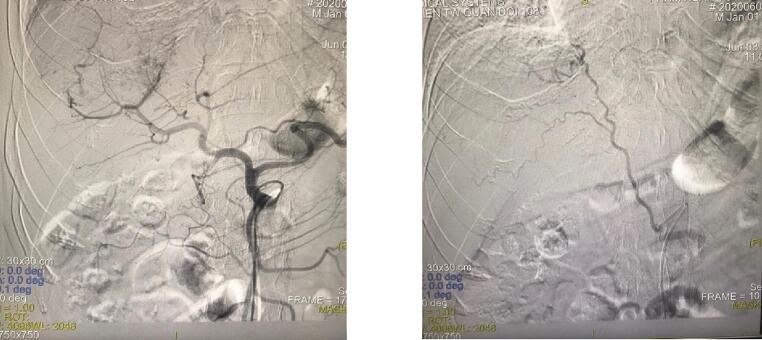
Right hepatic artery and right inferior phrenic artery.

The follow-up CT-Scan after 01 month showed there was residual tumor, so the 2nd TACE was indicated. The laboratory test before the 2nd TACE is summarized in Table 1. On angiography, the right hepatic artery and right inferior phrenic artery supplied a portion of the tumor. We successfully used doxorubicin, mitomycin, and cisplatin to chemo-embolize both the right hepatic artery (Fig. 2) and the inferior phrenic artery (Fig. 3).

**Table 1:** Laboratory tests results before the 2nd TACE intervention.

Blood tests	Results
Creatinin	101 μmol/l
AST	32 U/l
ALT	43 U/l
Total bilirubin	0.52 mg/dl
Albumin	36.1 g/l
INR	1.12

**Figs. 4, 5:**
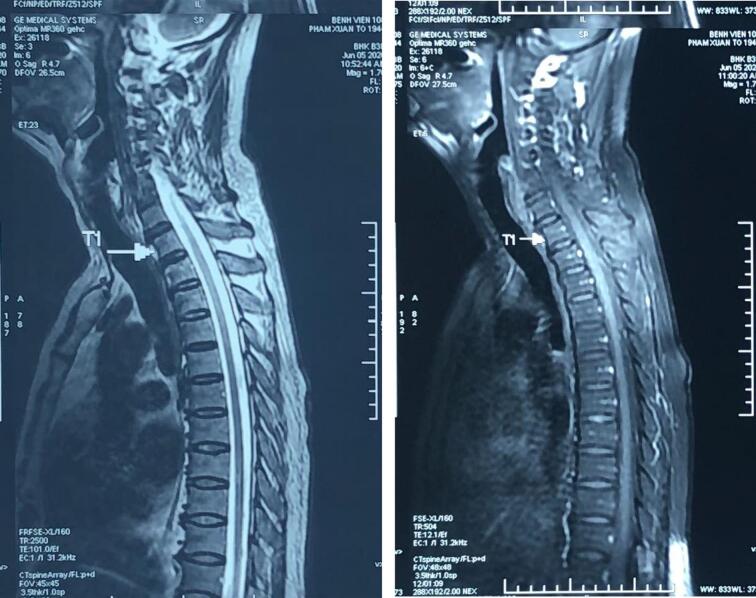
At the T1-T12 level, MRI showed increased intramedullary signal intensity.

Right after the intervention, the patient complained of acute paraplegia, a loss of somatic sensation in both lower extremities, the trunk below the sternum level, difficult urination, and dyschezia. Upon neurological evaluation, his lower extremity strength was 2/5 on the right and 3/5 on the left. With urinary retention was accompanied by tingling in the left anterior thigh and scrotum. There was no decrease in rectal tone, and the reflexes were bilaterally 2+. Both nerve conduction tests and electromyography were within normal ranges. The chemotherapy-induced localized vasculitis of the anterior spinal artery branches was identified as the patient's condition. Twenty-four hours following the TACE treatment, spinal magnetic resonance imaging (MRI) was conducted, and T2-weighted scans showed increased intramedullary signal strength at the T1-T12 level (Figs. 4, 5). Given the neurologic symptoms and indications, we hypothesized that TACE produced an ischemia lesion to the spinal cord. The patient was indicated high-dose steroids, which gradually lowered over one month. A rehabilitation program was also started while maintaining urine and feces using an enema and a Foley catheter. The motor strength remained the same, but the sensory deficiencies almost improved.

## 3 Discussion

HCC now is the leading cancer-related death in the world [1]. The principles of using embolization to treat these malignancies are provided by the predominant arterial blood supply of the tumors [1], [6]. With a median survival of more than four years, TACE is the first-line treatment for Barcelona Clinic Liver Cancer B patients (multifocal HCC without vascular invasion or extrahepatic dissemination, Child-Pugh class A-B, performance status 0) [1].

Despite TACE's less invasiveness and relative safe being its main advantages, TACE may also cause adverse effects [7], [8]. Rarely do TACE-related neurological problems develop, typically resulting from lipoidal embolisms in the central nervous system. Imaging studies can demonstrate that focal symptoms appear in the damaged vessel's distribution area. After TACE, spinal cord damage is an infrequent event with a prevalence incidence of 0.3 % but devastating effects. The hepatic artery injury or decreased flow at the prior TACE site, which causes collateral recruitment, can explain why spinal cord injury following TACE typically happens after the second or third session [4], [8]. It can occasionally result from accidental embolized spinal branches originating from intercostal or lumbar collateral arteries [9], [10]. About 6 to 8 h after the treatment, the clinical symptoms and indicators of spinal cord injury typically appear as paresthesias, reduced sensory function, paraparesis or paraplegias, and urine retention [4], [8]. In the initial phase, MRI findings are often normal; however, hyperintensities on T2-weighted images and focal cord enlargement are anticipated to appear after 1–2 days, and cord enhancement after gadolinium injection is expected to occur even later. The sensitivity and specificity of MRI in individuals with suspected acute spinal cord ischemia can be improved with repeated scans [11].

Our patient experienced bilateral lower extremities weakness, numbness in the left anterior thigh, and urine retention. The spinal cord injury is suspected, and damage to the T1–T12 spinal cord area can account for all these symptoms. The MRI result confirmed the diagnosis. The initial TACE was carried on with the right hepatic artery had been embolized completely. The right inferior phrenic artery involvement during the second TACE session caused this problem because it fed a portion of the tumor with blood.

The inferior phrenic artery is the most frequent source of extrahepatic collateral blood flow for HCC [12]. Usually emerging from the aorta above the celiac artery, the right and left inferior phrenic arteries each split into medial and lateral branches. They give most of the blood to the diaphragm and transmit a few branches to the adrenal glands, infrequently to the liver, and occasionally to the spleen. The medial branch connects to the musculophrenic and pericardiophrenic arteries and its counterpart on the other side. In contrast, the lateral branch connects to the musculophrenic and lower intercostal arteries. In our case, we hypothesize the embolism caused the infarction to the spinal cord travel through the connection between the lateral branches of the right inferior phrenic artery and the intercostal arteries, which supply the spinal cord through the anterior spinal artery. There are a few reports on the paraplegia brought on by TACE. A 45 year old male patient developed bilateral lower extremities weakness was reported by Tufail et al. [4] 8 h following TACE embolizing the right lower phrenic artery. In this instance, they proposed that anastomosis between the lateral branches of the right inferior phrenic artery and the intercostal arteries was the root of the paraparesis. The 11th and 12th intercostal arteries were used in a TACE procedure, according to one case in Chung et al.'s [6] series. Yet, their patients did not receive any specific prognostic information. Two examples of paraplegia that resulted from TACE and intercostal arteries were reported by Kim et al. [8]. In both cases, the complications happened in the 19th and 6th applications of TACE, respectively. In the first case, motor problems did not fully recover even two months after the injury, while sensory disorders did mend promptly. This indicates that the anterior spinal artery infarction brought on a spinal cord injury. The diagnosis of localized vasculitis was made in our patient because the comprehensive workup for paraparesis had been essentially unremarkable, with routine imaging, electromyography, and nerve conduction investigations. As a result, therapy with steroids led to the symptoms' remission. Neurological symptoms associated with TACE are among the few ischemia consequences and mostly are cerebral embolisms and rarely cause spinal cord ischemia [2], [8], [9]. There are no precise therapy recommendations. The level of spinal cord ischemia will determine the type of general medical care needed. In addition to rehabilitation techniques, great attention must be paid to skin, bowel, and bladder function [13].

## 4 Conclusions

In this case, we reported a rare complication with spinal cord damage following TACE. This case study demonstrates how TACE can have severe complications that can cause a lot of morbidities. A tailored therapeutic strategy, including consideration of a shunt and selection of the vessels utilized for the Lipiodol infusion prior to TACE, is crucial to achieving an optimal end outcome to avert these significant consequences.

## Informed consent

Written informed consent was obtained from the patient for publication of this case report and accompanying images. A copy of the written consent is available for review by the Editor-in-Chief of this journal on request.

## Provenance and peer review

Not commissioned, externally peer-reviewed.

## Ethical approval

This study was approved by Institutional Ethical Committee.

## Funding

The study did not receive external funding.

## Guarantor

Le Van Thanh.

## Research registration number

8869.

## CRediT authorship contribution statement

**Trung Hieu Le**: Methodology, Writing - original draft.
**Van Thanh Le**: Oversee the project, final approval of the manuscript.
**Van Quang Vu**: Methodology, Editing - original draft.
**Kim Khue Dang**: advising, editing English, and submitting the manuscript.
**Nguyen Hoang Ngoc Anh**: Collecting data.
**Dao Tien Duc**: Advising, reviewing the manuscript.

## Conflict of interest

We have no conflicts of interest to disclose.

## References

[1] Bruix J., Gores G.J., Mazzaferro V. (2014). Hepatocellular carcinoma: clinical frontiers and perspectives. Gut.

[2] Choi C.S., Kim K.H., Seo G.S., Cho E.Y., Oh H.J., Choi S.C., Kim T.H., Kim H.C., Roh B.S. (2008). Cerebral and pulmonary embolisms after transcatheter arterial chemoembolization for hepatocellular carcinoma. World J. Gastroenterol..

[3] XIE Feng, Feng X.U., YANG Jia-mei, Mengchao W.U. (2007). Analysis of serious complications of transcatheter arterial chemoembolization for the treatment of hepatocellular carcinoma. Journal of Medical Colleges of PLA.

[4] Tufail K., Araya V., Azhar A., Hertzog D., Khanmoradi K., Ortiz J. (2010). Paraparesis caused by transarterial chemoembolization: a case report. World J. Hepatol..

[5] Agha R.A., Franchi T., Sohrabi C., Mathew G., for the SCARE Group (2020). The SCARE 2020 guideline: updating consensus Surgical CAse REport (SCARE) guidelines. International Journal of Surgery.

[6] Chung J.W., Park J.H., Han J.K., Choi B.I., Han M.C., Lee H.S., Kim C.Y. (1996). Hepatic tumors: predisposing factors for complications of transcatheter oily chemoembolization. Radiology.

[7] Xia J., Ren Z., Ye S., Sharma D., Lin Z., Gan Y., Chen Y., Ge N., Ma Z., Wu Z., Fan J., Qin L., Zhou X., Tang Z., Yang B. (2006). Study of severe and rare complications of transarterial chemoembolization (TACE) for liver cancer. Eur. J. Radiol..

[8] Kim J.H., Yeon J.E., Jong Y.K., Seo W.K., Cha I.H., Seo T.S., Park J.J., Kim J.S., Bak Y.T., Byun K.S. (2010). Spinal cord injury subsequent to transcatheter arterial chemoembolization in patients with hepatocellular carcinoma. Dig. Liver Dis..

[9] Park S.J., Kim C.H., Kim J.D., Um S.H., Yim S.Y., Seo M.H., Lee D.I., Kang J.H., Keum B., Kim Y.S. (2012). Spinal cord injury after conducting transcatheter arterial chemoembolization for costal metastasis of hepatocellular carcinoma. Clin Mol Hepatol..

[10] Kim H.C., Chung J.W., Lee W., Jae H.J., Park J.H. (2005). Recognizing extrahepatic collateral vessels that supply hepatocellular carcinoma to avoid complications of transcatheter arterial chemoembolization. Radiographics..

[11] Alblas C.L., Bouvy W.H., Nijeholt GJ Lycklama À., Boiten J. (2012). Acute spinal-cord ischemia: evolution of MRI findings. J Clin Neurol..

[12] Kim H.C., Miyayama S., Choi J.W., Kim G.M., Chung J.W. (2023). Hepatocellular carcinoma supplied by the inferior phrenic artery or cystic artery: anatomic and technical considerations. Radiographics.

[13] Balami J.S., Chen R.L., Buchan A.M. (2013). Stroke syndromes and clinical management. QJM.

